# Physical Properties of Thermoplastic Starch Derived from Natural Resources and Its Blends: A Review

**DOI:** 10.3390/polym13091396

**Published:** 2021-04-26

**Authors:** Z. N. Diyana, R. Jumaidin, Mohd Zulkefli Selamat, Ihwan Ghazali, Norliza Julmohammad, Nurul Huda, R. A. Ilyas

**Affiliations:** 1 Fakulti Kejuruteraan Mekanikal, Universiti Teknikal Malaysia Melaka, Hang Tuah Jaya, Durian Tunggal 76100, Melaka, Malaysia; nurdiyanazakuan@gmail.com (Z.N.D.); zulkeflis@utem.edu.my (M.Z.S.); 2 Fakulti Teknologi Kejuruteraan Mekanikal dan Pembuatan, Universiti Teknikal Malaysia, Melaka, Hang Tuah Jaya, Durian Tunggal 76100, Melaka, Malaysia; ihwan@utem.edu.my; 3 Faculty of Food Science and Nutrition, Universiti Malaysia Sabah, Kota Kinabalu 88400, Sabah, Malaysia; norliza@ums.edu.my; 4 School of Chemical and Energy Engineering, Faculty of Engineering, Universiti Teknologi Malaysia (UTM), Skudai 81310, Johor, Malaysia; ahmadilyas@utm.my; 5 Centre for Advanced Composite Materials (CACM), Universiti Teknologi Malaysia (UTM), Skudai 81310, Johor, Malaysia

**Keywords:** thermoplastic starch, biopolymer, composite, food packaging

## Abstract

Thermoplastic starch composites have attracted significant attention due to the rise of environmental pollutions induced by the use of synthetic petroleum-based polymer materials. The degradation of traditional plastics requires an unusually long time, which may lead to high cost and secondary pollution. To solve these difficulties, more petroleum-based plastics should be substituted with sustainable bio-based plastics. Renewable and natural materials that are abundant in nature are potential candidates for a wide range of polymers, which can be used to replace their synthetic counterparts. This paper focuses on some aspects of biopolymers and their classes, providing a description of starch as a main component of biopolymers, composites, and potential applications of thermoplastics starch-based in packaging application. Currently, biopolymer composites blended with other components have exhibited several enhanced qualities. The same behavior is also observed when natural fibre is incorporated with biopolymers. However, it should be noted that the degree of compatibility between starch and other biopolymers extensively varies depending on the specific biopolymer. Although their efficacy is yet to reach the level of their fossil fuel counterparts, biopolymers have made a distinguishing mark, which will continue to inspire the creation of novel substances for many years to come.

## 1. Introduction

Plastic manufacturing and distribution across the world have been steadily increasing throughout time. Petroleum-based plastics are commonly employed as single-use plastics in our daily lives as they have become incredibly useful due to certain qualities, such as versatility, durability, flexibility, and toughness [1]. They are also very cheap in the market and easy to find in any grocery store. It was mentioned in the literature that, since the 1950s, the production of plastic had increased from 1.5 million tons to more than 300 million tons in 2015, which is almost 200 times more [2]. The rapid growth of plastic usage is due to the various types of plastic products currently found in the market, ranging from household, personal goods, and packaging to the manufacturing of construction materials. The extensive use of plastics has led to the excessive plastic waste currently present in the environment. In due course, this will create widespread global issues for the environment as well as for humans since the degradation rate of these materials takes a very long time, around 100 years, due to their hydrophobic properties and properties that efficiently avoid rapid microbial action [3].

Therefore, to overcome this issue, a change needs to be made by replacing the commonly used petroleum-based plastic with biodegradable plastic to sustain a better environmental condition for future generation and also to provide less plastic disposal techniques. Among biopolymers, starch is the one of promising possibility and alternatives to replace petroleum-based plastics, which is due to properties of starch that is completely biodegradable and are found to be abundant in nature. It is widely accessible in plants such as corn, cassava, potato, tuber, and many more. In plants, starch is stored in the granule packed form present in an amorphous and crystalline condition [4]. Due to great concern of environmental pollution, starch-based bioplastic known as thermoplastic starch (TPS) is commonly used today in packaging materials and some utilizing bio-fillers or fibre in the formulation to enhance the bio-based plastic. TPS is low in density, cheap, and has almost a similar strength value as petroleum-based plastic. Several studies on TPS have been widely and globally conducted for various sources of starch such as cassava, potato, and corn [5,6,7,8,9,10,11,12,13]. This review presents the recent developments of thermoplastics derived from starch and their potential in the industry. Starch modification is also highlighted, including blending with other naturally derived materials, which seem to further improve the mechanical and physical properties of the resulting bio-composite.

## 2. Biopolymer

As part of the awareness of global environmental issues concerning plastic waste, bio-based polymers are now promoted as alternatives for replacing petrochemical-based (fossil) polymers to supply increasing demand [9]. This initiative promotes the use of green environmental-based plants or animals in the production of bioplastic-based or bio-based in order to preserve and protect the environment. The worldwide interest for bio-based polymers has increased in recent years due to the desire to replace petrochemical-based polymer materials as well as the need to explore the innovative advances achieved in biotechnology [9]. Therefore, several initiatives have begun with the realization of the importance of producing more cost-effective and eco-friendly products, signifying the presence of numerous developments and production of bio-based materials [13].

**Figure 1:**
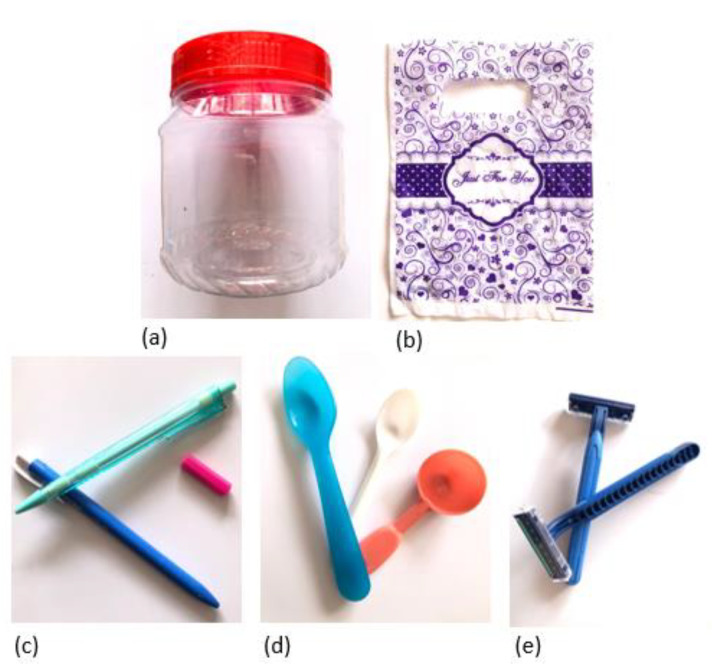
Biopolymer products: (**a**) food container, (**b**) plastic bag, (**c**) pens, (**d**) plastic spoon, and (**e**) shaving razor.

Many research works have been previously conducted on biopolymer products derived from natural sources. In general, the animal-based biopolymer refers to the gelatin, which can be extracted from the skin and bone of porcine, bovine, poultry, and fish [14,15,16]. Meanwhile, the plant-based biopolymer can be characterized as starch, cellulose, hemicellulose, and lignin. Starch is the main component of the biopolymer structure, which acts as a matrix, and, with the addition of plasticisers, the flexibility and processability of the resulting thermoplastic starch (TPS) are further enhanced. Many biopolymer products have utilized natural fibres as fillers in their composition. Natural fibres, mainly from agricultural industries, were collected and tested with TPS to become bio-composite materials. Different types of fibres obtained from diverse agro-industrial residues have been investigated, such as bamboo bagasse [17], cassava bagasse [18,19], grape stalks [20], sugarcane, orange, cornhusk bagasse [3], cogon grass fibre [21], and kraft fibre [22]. Figure 1 presents the bio-based product applications that have been developed and examined.

## 3. Starch

Starch is the main component of the biopolymer structure that belongs to the polysaccharides group and is considered the second most crucial renewable sources after cellulose. It is inexpensive and completely biodegradable, generating great interest to make it into a component of bioplastic. Starch is known to be a versatile material since it can be converted into chemicals such as ethanol, acetone, and organic acids used in synthetic polymer production, such as polylactic acid (PLA) [23] and can also be converted to TPS with the aid of plasticizer under a shear temperature condition [1]. Pure starch is white in appearance. It is a tasteless and odourless powder that has been one of the most abundant natural polysaccharides synthesized from plants [17] with starch worldwide production of more than 50 million tons per year [24] and cassava starch is the main contributor among other sources where it could produce for about two to four times more than that of yam bean, taro, and about 10 times higher than that of sweet potato [25]. However, Bergthaller & Hollmann [26] reported that corn starch is the crop most produced globally for about 80% of total world starch production and followed by wheat, tuber, and cassava where United States dominantly cultivates the highest starch production besides the European Union (EU) and other countries. Starch sources are derived from plants (such as corn, potatoes, cassava, and wheat) and stored as carbohydrates [27]. Starch is composed mainly of two homopolymers of D-glucose amylase, a mostly linear α-D(1, 4′)-glucan, and branched amylopectin. They have the same backbone structure as amylose but with many α-1, 6′-linked branch points [28].

The hydroxyl group in the starch structure exhibit the reaction of oxidation and reduction processes as well as the formation of hydrogen bonds, ethers, and esters. Native starches contain about 70–85% amylopectin and 15–30% amylose. The presence of amylopectin is mainly responsible for the crystallinity of the starch granules [29]. However, Domene-López et al. [4] and Sahari et al. [30] reported that the crystallisation ability is influenced by the molecular weight and phosphate monoester content in starch as these features contribute to lower starch chain rearrangement. It was stated that starch with high amylose content can have low molecular weight and a relatively more linear structure than those with a high content in amylopectin. Thus, starch that contains a high amylose value could facilitate further crystallisation processes as compared to starch with high amylopectin content. Relative molecular weight of these molecules depends on the botanical origin of the starch and might influence the final bio-composite properties’ application [31].

## 4. Thermoplastic Starch

Neat starch displays some disadvantages due to its high solubility in water, brittleness, poor melting point, and lower mechanical properties as compared to materials made of synthetic polymers. To enhance the properties of starch, various physical or chemical modifications, such as plasticisation, blending, derivation, and graft copolymerisation were investigated [1,17,32,33,34]. TPS is made by applying mechanical and thermal energy onto the starch granules by adding plasticizer. Plasticisers play a vital role in the preparation of thermoplastic starch as they improve starch behaviour by reducing internal hydrogen bonding in between the polymer chain while increasing free volume. This, in turn, increases flexibility and processability and promotes molecular chain mobility [32,35]. The effectiveness of plasticisers depends on the similarity of the polymer used.

Plasticisers can be found in several forms, such as glycerol, sorbitol, urea, fructose, sucrose, and glycol [36]. However, the most commonly used plasticisers are from the polyol group, namely, glycerol and sorbitol [32]. Several research works have been recently conducted to explore certain ionic liquids (ILs) as new starch plasticisers. Ionic liquids include 1-ethyl-3-methylimidazolium acetate ([emim^+^][Ac^−^]) [35,37] and 1-butyl-3-methyllimidazolium chloride ([bmim^+^][Cl^−^]) [33]. Besides, a recent study employed fried sunflower oil as a plasticiser in thermoplastic starch composites. An improvement is shown in starch-based material properties, proving to be the most environmental solution for bio-composites [38].

A comprehensive study was conducted by Demash and Miyake [35] regarding the effect of four different types of plasticisers on anchote *(Coccinia abyssinica)* starch film. Glycerol, 1-ethyl-3-methylimidazolium acetate, sorbitol, and tri-ethylene glycol were utilised at concentrations of 30% and 40% *w*/*w* in the starch mixture. The thermoplastic film samples were then dried overnight in the oven at 50 °C before they were kept in a desiccator for at least two days for film characterisation. The prepared film was transparent, homogenous, and flexible. The same sample preparation was reported by Reference [32]. The obtained results revealed that there is an increase in thickness for all samples as plasticiser concentrations increased. The sample with 40% tri-ethylene glycol plasticiser exhibited the highest film thickness with a value of 0.26 mm, thus, reflecting the lowest density value of 0.88 g/cm^3^ among other samples. It was found that the water solubility of glycerol and tri-ethylene glycol plasticised film had reduced from 32.57 g/cm^3^ to 20.97 g/cm^3^ and 34.19 g/cm^3^ to 18.92 g/cm^3^, respectively. According to Reference [1], the same behaviour was observed when glycol was used as a plasticiser. This may be due to the formation of strong hydrogen-bonds with starch, which restrain water molecules to combine with plasticized anchote starch. Plasticisers presented an improvement in the film since it had less affinity to water. However, sorbitol and 1-ethyl-3-methylimidazolium acetate plasticised film displayed a different result of water solubility. Water solubility increased as the plasticiser concentration increased.

Nevertheless, biodegradability of the film plasticized with ILs does not specify in the study which could be one of the interesting points that need to be highlighted. Across the journal available, there is also limited research that working on the biodegradability of ILs as a plasticizer. However, Rynkowska et al. [39] reported that ILs is non-toxic liquid and is considered as a green alternative to substitute phthalates, commonly used as plasticizers in synthetic material, which is primarily to soften polyvinyl chloride and is harmful to humans. Besides, ILs portray unique properties such as non-volatility, low toxicity, easy to handle, inflammable, and goods in ions conductivity [40]. An interesting study made by Sudhakar and Selvakumar [41], investigated on biodegradable properties of the plasticized chitosan and starch blends electrolyte film, found lithium perchlorate (LiClO_2_) using an activated sludge degradation method. The samples are pre-weighed before being immersed in the sludger under an aerobic condition for 5, 10, and 15 days and taken out to be weighed again after being cleaned and dried in an oven at 75 °C. The result obtained showed a degradation range between 6.2–16% for 0, 0.5, 1.0, 1.5, and 2.0 of lithium perchlorate. It presents that the percentage of weight lost increased as the amount of lithium perchlorate was added. This might be attributed to the presence of lithium perchlorate that promoted free volume in the polymeric matrix and, thus, enhance the biodegradability rate [41]. On that note, it can be concluded that ILs are safe for the environment and also for humans.

### 4.1. Thermoplastic Cassava Starch

In the production of biopolymers, starch is mainly used as a matrix or resin in bio-composite structures. Throughout past decades, numerous types of natural starch have been investigated, such as cassava starch, corn starch, sugar palm starch, and much more. However, cassava starch is the highest contributor in terms of productivity yield compared to other sources of starch [25]. Some research works reported cassava starch as tapioca starch, depending on the author. The name ‘cassava’ is generally applied to the roots of the plant, whereas tapioca is the name given to starch and other processed products [42]. Table 1 presents the application of cassava starch as a thermoplastic in bio-composites.

Recent studies have been accomplished on polymer composites of treated oil palm mesocarp fibres (OPMF) and thermoplastic cassava starch properties prepared by using the screw extrusion rheometer method [6]. Two types of OPMF were studied: raw and alkaline treated OPMF mixed with thermoplastic cassava starch (TPS) at different weight loadings (5–20% for raw and 5–20% for alkaline treated OPMF). It was stated that OPMF is composed of a large source of lignocellulosic material and can be used as bioethanol production and as reinforcement in polymer composites [43]. The addition of OPMF was expected to lead to an increase in tensile strength due to the great adhesion between the fibre and matrix, contributing to their chemical affinity. Chemical treatments, such as alkali treatments, were employed to enhance the fibre-matrix interaction by removing lignin, oils, waxes, and silica, which have collectively exhibited a fibrillation process during the removal of hydrophilic components.

**Figure 2:**
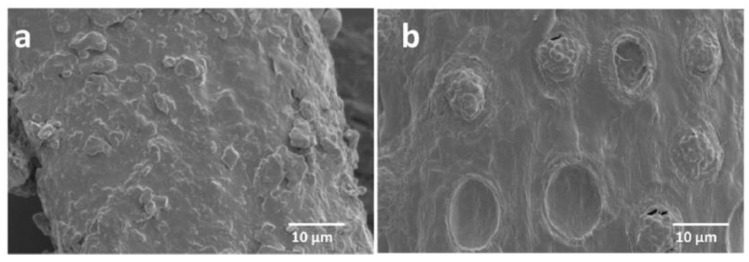
Scanning Electron Microscopy (SEM) of: (**a**) raw oil palm mesocarp fibres (OPMF), and (**b**) alkaline treated OPMF [6].

**Figure 3:**
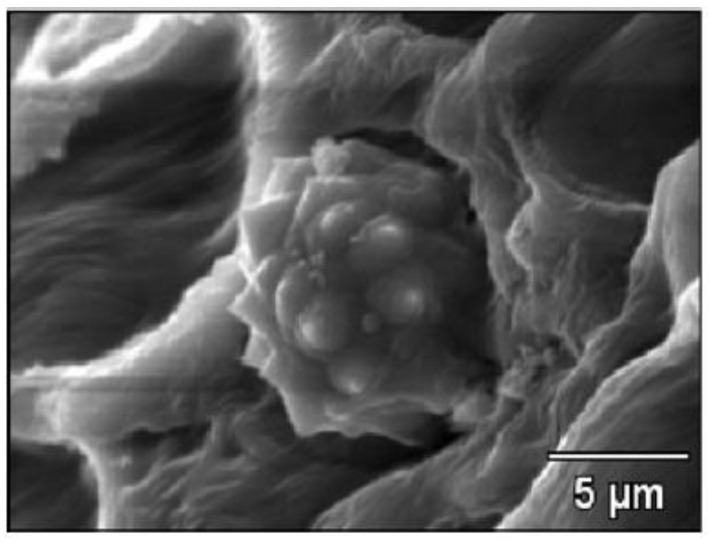
Energy Dispersive Spectroscopy (EDS) of OPMF [6].

**Figure 4:**
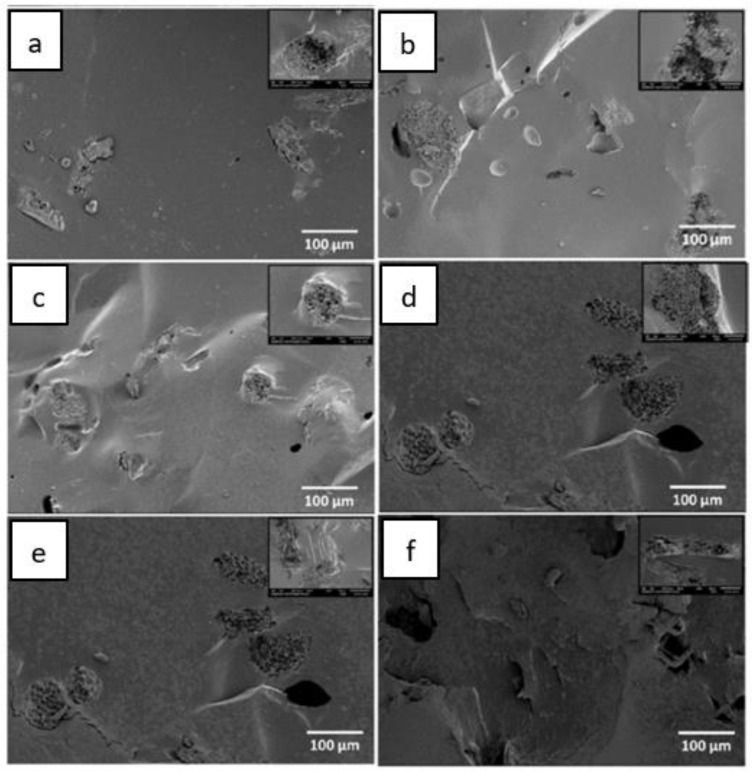
Micrography of (**a**) thermoplastic starch (TPS) 5% raw fibre, (**b**) TPS 5% alkali treated fibre, (**c**) TPS 10% raw fibre, (**d**) TPS 10% alkali treated fibre, (**e**) TPS 20% raw fibre, and (**f**) TPS 20% alkali treated fibre [6].

The obtained results from the Scanning Electron Microscopy (SEM) for raw and alkaline-treated OPMF and Energy Dispersive Spectroscopy (EDS) are presented in Figure 2 and Figure 3. Figure 3 displays the silica removal, observed by craters on the surface, after alkaline treatment was conducted. A few thorny structures had adhered onto the surface, indicating the presence of amorphous substance or silica, which remained in the OPMF. The raw fibres presented an average length of 440–1000 μm and a diameter of 100 μm. These results validate the previous research conducted by Reference [44], which stated the influence of alkaline treatment on the removal of silica. Figure 4 displays a cross-section of TPS composites with raw and alkali-treated fibres. It is clear that the polymer matrix covered the fibres and promoted a strong adhesion between them. The fibre-polymer interaction was favoured by the residual silica presence, whose oxygen atoms interact very strongly with the TPS hydroxyl groups.

### 4.2. Thermoplastic Corn Starch

Corn starch is seen as a promising source of biopolymer matrix that is environmentally friendly and suitable for replacing petroleum-based plastics. Pure corn starch is similar to other natural starches, which have some drawbacks such as high-water sensitivity and poor mechanical behaviour. Thus, modification of starch is required by introducing plasticisers in the mixture. Plasticised starch properties have enhanced abilities. Thermoplastics from corn starch have been widely explored and various modifications were accomplished to improve the properties of the resulting TPS. The recent studies conducted had utilised waxy corn products in their works. It was reported that waxy corn produces starch that is nearly 100% amylopectin [45]. Table 2 presents the recent studies conducted on thermoplastics from corn starch composites.

**Figure 5:**
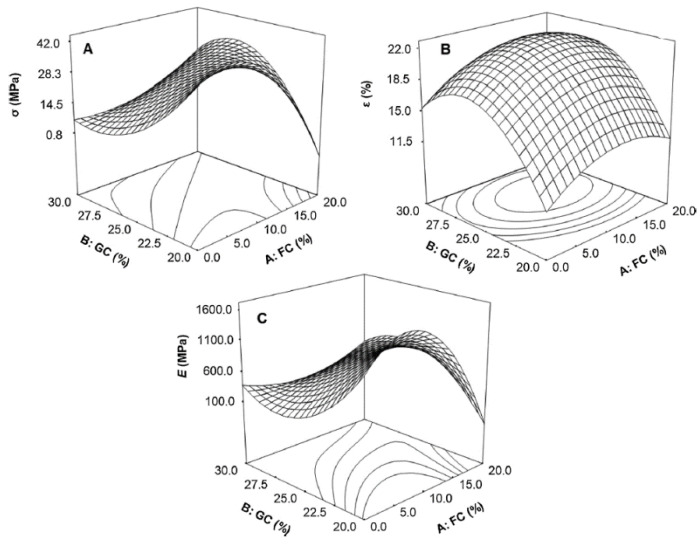
Effect of acetylated sugarcane fibre content (FC) and glycerol content (GC) on: (**A**) tensile strength (σ), (**B**) elongation (ε), and (**C**) young modulus of acetylated starch-based bio-composites reinforced with acetylated sugarcane fibre [46].

Fitch-Vargas et al. [46] studied the modification of corn starch bio-composites reinforced with sugarcane fibre. A process called acetylation of starch and fibres was performed, resulting in the formation of acetylated corn starch bio-composites reinforced with acetylated sugarcane fibre and led to enhancement of the resulting bio-composite’s behaviour, such as improved processability and compatibility [51]. Different loadings of acetylated sugarcane fibre contents (FC, 0.0–20.0%) and glycerol contents (GC, 20.0–30.0%) were examined in this work. Figure 5 and Table 3 presents the mechanical test results of the samples. With reference to the tensile result of synthetic polymers, polystyrene (30–55 MPa) and polypropylene (25–40 MPa), it was found that the highest tensile value (35 MPa) is achievable with the composition of GC 24–28% and FC 10–15%. This was attributed to the effect of glycerol opening the polymeric 3D structure, which allowed fibre incorporation. Besides that, the presence of acetyl group may function as a spacer between the starch chain for cellulose fibre to fill in and improve the bonding interaction [53]. It was observed that exceeding 12% FC leads to a decline in strength due to poor fibre distribution. The highest elongation value was obtained when 24–28% GC and 5–15% FC were mixed together. This reflects on the great number of linkages formed due to the fibre’s surface chemical treatments conducted, which allowed the active fibres’ hydroxy group to react with the matrix. Thus, this enhanced the mechanical interlocking on the rough surface, forming stronger secondary bonds.

**Figure 6:**
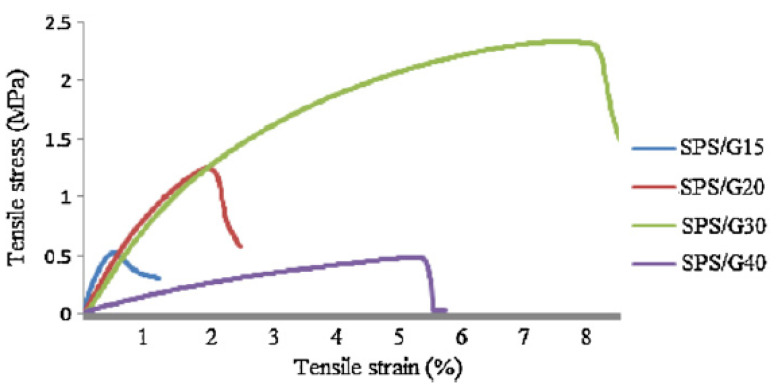
Tensile stress-strain of plasticized sugar palm starch (SPS) [1].

On the other hand, a moisture adsorption test is carried out to identify the sample’s affinity toward the surrounding moisture condition. The samples are placed into two different relative humidity (RH) desiccators conditions at 53% RH and 100% RH. Weight gained among the samples are taken until constant weight are found, indicating that the samples have reached an equilibrium state. As per Figure 6, the finding shows that, as the GC increased, the moisture absorption (MA) value increased where the lowest MA are obtained at GC less than 25% throughout the FC range. The same results are also found in a water solubility (WS) test. This could be attributed to the formation of a stronger hydrogen bond between the polymeric matrix and has restricted the entrance of surrounding moisture. Besides, this might be due to the incorporation of fibre that is low hygroscopic in nature and has further enhanced the barrier properties. However, MA and WS are seen to increase considerably when the GC loading increased to more than 25%. It is reported that higher GC has the ability to reduce the hydrogen bonding and, therefore, has led to an increase of the intermolecular spaces due to material swelling and weakening of the bonding forces between the water molecule and hydrophilic functional group. In contrast, others have reported on the reduction of MA by increasing glycerol in plasticized starch and reduction of MA by increasing glycerol and fibre content in the resulting bio-composite (Sahari et al. [1] and Jumaidin et al. [25], respectively). They stated high glycerol and fibre contents incorporated have shown better resistance toward moisture due to stronger hydrogen formed, which restrained the combination between the water molecule and the sample. The journal focuses on how GC may affect the result and lack of more explanation in terms of an FC interaction that also has many contribution effects toward the obtained results.

### 4.3. Thermoplastic Sugar Palm Starch

To date, numerous research works utilising sugar palm starch in the production of bio-composite materials as an alternative to petroleum-based polymers have been accomplished. Natural starch from sugar palm trees was found to be abundant within the tree trunk [54]. Many authors had mentioned the multifunctional uses of the sugar palm tree. The extraction of sugar palm starch is usually done on unproductive trees [29]. Whether the tree is considered mature enough or due to excessive microbial attacks, the tree is unable to normally grow in a good condition. Thermoplastic starch derived from sugar palm trees was successfully developed in the presence of biodegradable glycerol as a plasticiser. Table 4 shows the utilisation of sugar palm starch (SPS) as a matrix in bio-composites.

Sanyang et al. [29] briefly explained the processes involved for extracting sugar palm starch from the sugar palm trunk. The starch extraction process starts with the unproductive sugar palm trees that are first cut down. Next, the tree trunk is divided lengthwise, and all the woody fibres are removed. The inner core stem is then cut out to obtain the mixture (off-white in appearance). The collected fibre and starch mixture subsequently undergo a washing process using water, and then thoroughly kneaded by hand. Next, the mixture is sieved, allowing water and starch granules to flow through. The filtered mixture is left to allow granule suspension. The white powder is then left in the open for a specified time. Finally, the white powder is dried in an oven for two days. In this research, it was reported that one sugar palm tree can yield 50–100 kg of starch.

Thermo-mechanical studies have been conducted by Sahari et al. [1] in an attempt to produce bioplastic using SPS as a composite matrix at different glycerol loadings from 15–40 *w*/*w*%. The results from this work revealed that, from the fracture surface analysis, as the glycerol content increased, acquiring a smooth surface also increased. At 15 *w*/*w*% glycerol loading, a brittle surface was the outcome, while an exceptionally smooth surface appeared at 40 *w*/*w*% glycerol loading. The glycerol, which acts as a plasticiser, effectively reduced the internal hydrogen bond while increasing the intermolecular spacing. This resulted in the appearance of a smooth surface.

In terms of the product’s mechanical properties, as illustrated in Figure 6, it was found that SPS/G30 and SPS/G40 showed the typical pattern of rubbery starch plastic materials due to the linear increase in tensile stress at a low strain, followed by the curve toward the strain axis until failure occurred. The addition of plasticisers at 30 wt% and 40 wt% overcame starch brittleness and improved flexibility.

## 5. Thermoplastic Starch Blends

Modifying thermoplastic starch can be accomplished through blending, which widens its application at lower cost. Compatibility of blends must be tested to examine the enhancement of the composite’s properties such as water resistance, high tensile strength, and high tensile modulus. Blending is also important for modifying bioplastics in terms of biodegradability, enabling them to decompose easily through natural processes so as to cater to the issue of limited disposal technology. Starch blending with a polar polymer containing hydroxyl groups such as polyvinyl alcohol, ethylene copolymer, and partial hydrolysed vinyl acetate have been prepared since the 1970s [23]. Blending is one of the most promising alternatives to make starch useful as a polymer in the replacement of other plastics, and the fast progress occurring in this field is attested by several reviews that have been recently published [59,60,61,62,63,64].

### 5.1. Starch/Polyvinyl Alcohol (PVA)

PVA is a synthetic biodegradable polymer that has the advantages of good film formation, strong conglutination, high thermal stability, and good gas barrier properties [65]. PVA is manufactured by the polymerisation of vinyl acetate monomer into polyvinyl acetate (PVAc), followed by the hydrolysis of the acetate groups of PVAc to PVA [64]. PVA is one of the major polymers used in the industry and, therefore, huge amounts of PVA are produced yearly. The world production of PVA is about 650,000 tons per year [64]. The presence of PVA in a blend increases the mechanical strength, water resistance, and weather resistance of the blend [65]. Gelatinisation is the most common method of blending starch with PVA.

The compatibility of PVA and starch enables them to form a continuous phase at blending [28] even though the properties of the blends deteriorate as starch content rises, causing phase separation during blend preparation. To improve the compatibility between PVA and starch, the addition of suitable plasticisers, cross-linking agents, fillers, and compatibilizers were examined [65]. Both PVA and starch can be plasticised into a thermoplastic material, regularly using the casting method and glycerol in an aqueous medium [66]. Both starch and PVA are biodegradable in several microbial environments. However, the biodegradability of PVA depends on its degree of hydrolysis and its molecular weight [63]. Both PVA and starch can be plasticized into a thermoplastic material, regularly using the casting method and glycerol in an aqueous medium [66]. Both starch and PVA are biodegradable in several microbial environments. However, the biodegradability of PVA depends on its degree of hydrolysis and its molecular weight [67].

### 5.2. Starch/Poly Lactic Acid (PLA)

PLA, a biodegradable polyester produced from renewable resources, is used for various applications such as biomedical, packaging, textile fibres and technical items. PLA is industrially obtained through the polymerisation of lactic acid through the ring opening polymerisation (ROP) method [62]. PLA is, by far, the most commercially developed, reaching an annual production volume of approximately 200,000 tons [61]. However, PLA is not environmentally biodegradable as it requires a proper composting facility that applies a heating temperature of 60 °C and it must also be exposed to special microbes that will digest and decompose the material. However, Hatti-kaul et al. [61] reported that PLA is composted of 100% L-lactide unit that takes 110 weeks to degrade, which can be reduced dramatically up to 10 weeks while adding 50% D-lactides unit and can further reduce the degradation time to 3 weeks when 25% glycolic acid is added in the formulation. Some other disadvantages of PLA include low flexibility, ductility, and impact resistance.

To improve PLA’s flexibility and impact resistance, numerous plasticisers are incorporated, such as poly (ethylene glycol), glycerol, glucose monoesters, citrate esters, and oligomers [68]. TPS, as a blend component for PLA, offers important advantages in terms of cost, properties, and biodegradability [62]. The hydrophilic characteristics of starch and the hydrophobic features of PLA cause low miscibility between the two compounds. For this reason, good melt-blending techniques and the addition of compatibilizers are required to increase a successful interaction, i.e., amphiphilic molecules or coupling agents [62]. Poly (hydroxyester ether), methylene diphenyl di isocyanate (MDI), PLA-graft-(maleic anhydride), PLA-graft-(acrylic acid), PLA-graft-starch, and poly(vinyl alcohol) are all used as compatibilizers in this blend [69].

### 5.3. Starch/Polybuthylene Succinate (PBS)

PBS can be found commercially as a thermoplastic-based polymer, which provide a high degree of crystallinity and takes a long time to degrade. PBS is a synthesis of polycondensation succinic acid and 1,4-butanediol where succinic acid can be obtained from either a monomer derived through a petroleum system or bacterial fermentation. Meanwhile, 1,4-butanediol is derived from fossil fuel extraction from formaldehyde or acetylene [70]. PBS provides improvement in a composite characteristic with strong impact strength, better chemical resistance, and high thermal stability [65]. Incorporation of starch blends with PBS has produced more flexibility and elasticity to the resulting material, which could be promoted as alternative food packaging. The blends could speed up degradation time due to the presence of starch that contains hydrogen bonding and is able to promote free spaces for degradation to occur [71]. However, Hatti-kaul et al. [61] stated that the addition of long chain dicarboxylic acid in the PBS homopolymer will form polybutylene succinate adipate (PBSA) in which its degradation time is less than PBS. PBSA is an aliphatic thermoplastic copolymer. PBSA annual production is estimated around 97,000 tons and is commercially available as a thermoplastic polymer [61].

## 6. Thermoplastic Starch Incorporated with Natural Fibre

### 6.1. Natural Fibre

The implementation of natural fibre as a promising alternative for replacing synthetic fibre in the composite industry has been increasing in the past decades. Synthetic fibres such as glass, carbon, and aramid reinforced with polymer matrix materials provide advantages of high stiffness and strength-to-weight ratio in numerous applications as compared to conventional construction materials [72]. Changes from the dominant usage of synthetic fibre to natural fibre indicate the rise of awareness among people around the world, regarding the negative environmental impact that synthetic fibre brings, which may disturb human health conditions. The utilisation of natural fibre provides advantages as compared to synthetic fibre in terms of favourable tensile properties, reduced health hazard, acceptable insulating properties, low density, and decreased energy consumption [27].

The production of synthetic polymers utilise a large quantum of energy, which produces environmental pollutants during the production and recycling of synthetic composites [72]. Table 5 illustrates a comparable study between natural and synthetic fibres, clearly displaying renewable, recyclable, and biodegradable properties as part of natural fibre properties. It should be noted that natural fibre properties vary depending on the source of fibre itself, including it species, environmental climate condition, geographical location, the process for preparing the fibre, etc.

**Figure 7:**
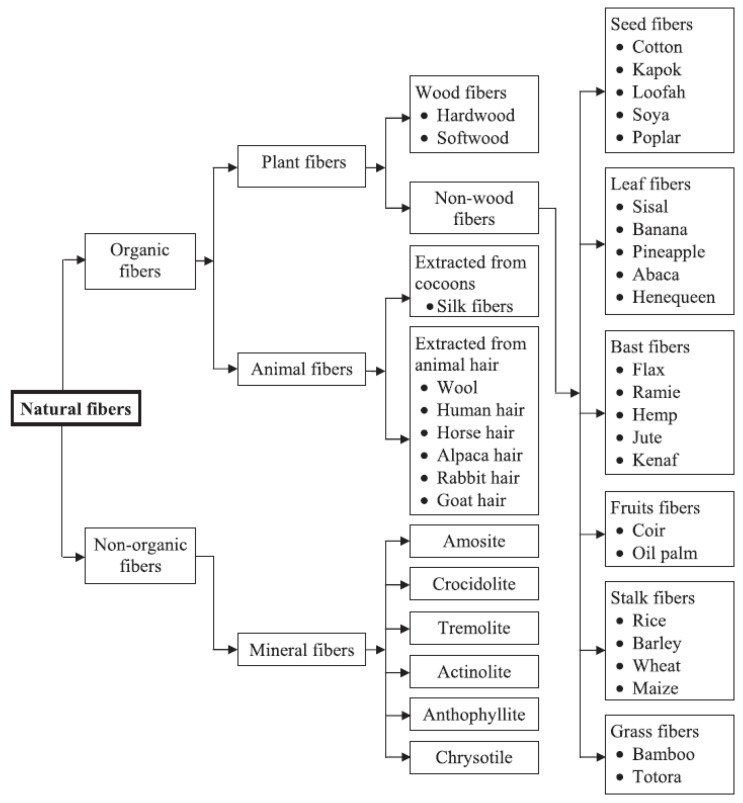
Classification of natural fibre [72].

Natural fibres reinforced composites are also applied as fillers in a matrix to provide high strength and stiffness on a weight basis to a composite product [73]. Fibre reinforcement added to the matrix phase (either natural resin, thermoset, and thermoplastic polymers) will act as a glue or binding agent to the composite formation [74]. The natural resin mentioned includes wheat starch, corn starch, potato starch, etc. [27]. Thermoset polymers include epoxy, polyester, and phenolics [74], and thermoplastic polymers include polycarbonate, polyvinyl chloride, and nylon [75]. Natural fibres are classified based on their origin in nature, either from plants, animals, or minerals. Figure 7 presents the classification of natural fibres. Most natural fibres come from plants and are composed of cellulose, thus, making the fibre hydrophilic in nature [17]. They are also called lignocellulosic fibres since their cellulose fibrils are embedded in the lignin matrix [29].

### 6.2. Cassava Bagasse Fibre

Cassava, also known as *Manihot esculenta C.*, is a root and tuber crop found in many tropical countries. Therefore, it is used as the main source of food. It is estimated that cassava could have a yield potential of up to 17,000 kg of starch per hectare per year if it is cultivated in a suitable environment with organised farming practices [25]. Cassava is ranked as the fifth most widely used starch source in the world and the third among food sources consumed in tropical countries [18]. Cassava bagasse is a fibrous solid residue obtained as a by-product of the industrial cassava starch production, which has undergone the separation of starch and fibre. It was reported that cassava bagasse residual fibre may consist of 38% cellulose and 37% of hemicellulose and lignin [76]. The cultivated cassava tuber for around 250–300 tons can yield a high moisture content of by-products (around 1.6 ton of solid peels and 280 tons bagasse). These solid wastes are generally discarded in the environment without any treatment [19]. This alarming issue has led to the awareness of utilising agricultural waste by converting them to other useful materials.

Many studies had focused on exploiting waste by utilising cassava bagasse as a filler or reinforcing agent to enhance the strength and mechanical properties of material products. This is especially conducted for the production of bio-based or bio-degradable materials, which have been steadily increasing. These by-products are low in market value and possess good properties with a wide range of applications, such as reinforcing agents or in the production of organic acid, bio-degradable packaging, nano particles, nanofibres, ethanol, bio-fuel, lactic acid, etc. [76]. Paula et al. [18] had shown that, when cassava fibres were added to corn starch, the film strength improved by up to 37.5%. This was attributed to the good intermolecular interaction between the starch and reinforcement. The elongation at break also reduced due to the possible agglomerates formed within the films. The same results were obtained by García et al. [19], verifying an increase in tensile strength as well as a decrease in elongation at break when fibres were added to the starch.

### 6.3. Sugarcane Fibre

Recently, the valorisation of sugarcane waste for low-cost building products has been increasing due to its high potential. Since there is a vast global production of sugarcane, especially in some tropical and subtropical developing countries, high amounts of sugarcane fibres are produced as by-products [77]. Fortunately, this provides numerous job vacancies with regard to this industry. It was reported that the current largest sugarcane world producer is Brazil where its yearly production exceeds 640 tons [78]. Previously, India was the largest with a yearly production of more than 100 million tons of bagasse [79]. Sugarcane bagasse is the residue obtained from the sugarcane industry after extracting the sugarcane juice. It has also become a source of fuel [46]. This by-product can be a source of biomass products and, therefore, has caught great research attention due to its vast availability, ecological features, and renewable characteristics found in the sugarcane fibre. It is seen to have great potential as a bio-composite product and can be used as a reinforcing agent.

With regard to the incorporation of natural fibres such as sugarcane into starch-based composites, numerous studies have reported and demonstrated significant improvements in the resulting bio-composite products. Fitch-Vargas et al. [46] stated that the addition of sugarcane fibre in the composite mixture has enhanced the product’s tensile strength as well as reduced water affinity, attributing to the strong interaction adhesion between the matrix and filler. However, Farias et al. [80] mentioned that this is not always the case since some modifications on the fibre itself may be required depending on the type of collected sugarcane. The discrepancies in the obtained results are influenced by the species of sugarcane, the age of sugarcane, and the surrounding climate. Hernández-olivares et al. [81] utilised sugarcane as a reinforcing component in the construction of cement, Ordinary Portland Cement (OPC), reporting its potential of improving composite durability. The authors demonstrated that incorporating reasonably high-volume fractions of sugarcane bagasse can produce feasible manufacturing composite materials for building construction since they exhibited enhanced physical and mechanical properties.

### 6.4. Bamboo Fibre

Growing interest on modification of surface fibre have been implemented to increase the reinforcement strength for the bio-composite. Surface modification treatments can be classified into three categories: chemical, physical, and biological [82,83]. One chemical method most used for the modification of vegetable fibres is mercerisation. This method involves modifying the structure and chemical composition of plant fibres using an aqueous solution of sodium hydroxide (NaOH) [84]. With this treatment, the removal of surface impurities and the occurrence of fibrillation are possible by obtaining a fibrous material with greater surface area and smaller diameter. This produces an increase in the fibre’s tensile strength and mechanical properties for the resulting composite. A physical process is the application of a cold plasma treatment that is considered a promising environmental method utilizing non-harmful gases, such as methane, argon, or helium [80]. This method allows the formation of free radicals and the polymerisation of the material. The plasma treatment acts on the chemical structure of fibres and their crystallinity index. Nevertheless, ozone treatment has emerged as an eco-friendly method for surface modification [85]. It is used to oxidize the lignin to stimulate the reactivity through the increase of hydroxyl group content and to produce low molecular weight of the soluble compound.

**Figure 8:**
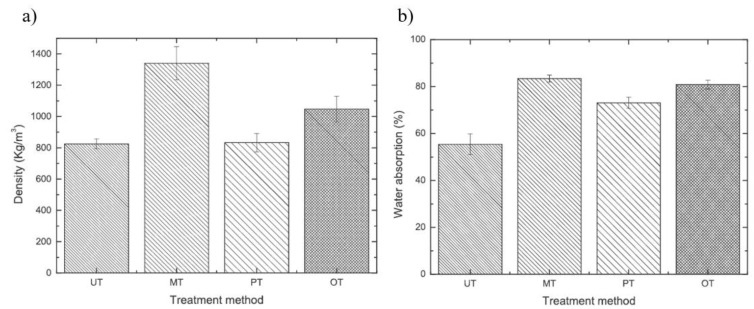
Effect of treatment on: (**a**) the density of bamboo fibre and (**b**) water adsorption of bamboo fibre [34].

The influence of surface treatments on bamboo fibres as reinforcement agents in TPS were conducted by Jhon et al. [34]. In this study, three different treatments were applied: the mercerisation treatment (MT), which modifies the structure and chemical compositions using sodium hydroxide (NaOH), the cold plasma treatment (PT), which conducts physical modifications using methane or helium gas, and the ozone treatment (OT), which produces soluble compounds low in molecular weight by oxidising lignin and hemicellulose. From Figure 8, it was found that the MT samples displayed a significant increase in density value (about 60%) as compared to the other treatments. As reported by Campos et al. [6], the alkaline treatment causes the removal of large parts of the amorphous substances, which includes lignin, polysaccharides, and waxes, resulting in the low molecular weight of the sample. The PT sample showed no significant difference and almost the same result was obtained when compared to untreated bamboo fibre (UT). For the water adsorption test, all treated samples exhibited an increase in the water adsorption percentage. Some origins of fibre that are hydrophobic in nature were associated with the presence of lignin, hemicellulose, and pectin [83]. Alterations to the substance composition had caused the formation of new behaviours, such as an increase in hydrophilic properties.

## 7. Application of Starch-Based Biopolymer

The development of starch blending is more interesting since it can substitute an older material while exhibiting the same properties. Table 6 highlights the main applications of a few widely used starch-based biopolymers. Biodegradable packaging applications have drawn remarkable attention for biopolymer utilisation in comparison to other areas because they are higher in significance. Biodegradable plastics are increasingly drawn toward addressing environmental impact in several particular applications, such as single-used plastic, greenhouse gas (GHG) emissions, and overload of plastic waste. These new implementations and innovations of bio-based plastics shown on increased awareness toward a better sustainable environment in the future, as they provide less of a disposal technique and degrade easily.

Sahari et al. [1] discovered on the potential of plasticized sugar palm starch (SPS) in a biodegradable packaging application. Unlike other starch, such as corn, cassava, or potato starch, sugar plam starch is a passed decade discovered on its true potential that is mostly as par as other starch. With the addition of glycerol as a plasticizer into SPS, it produced a biocomposite under a high temperature condition. The obtained mechanical result shows an increment as the glycerol content increases up to 30 *w*/*w*% with a maximum tensile value of 2.42 MPa. Besides, a reduction of transition temperature and water absorption as glycerol are added, indicating that the biocomposite has reduced its brittleness as glycerol loading increased. Meanwhile, Sanyang et al. [32] studied the different plasticizer affect SPS mechnical properties in an attemp of development biofilm, utilizing sorbitol, glycerol, and glycerol-sorbitol in varying conditions (0, 15, 30, and 45 *w*/*w*%). The results collected show the storage modulus of plasticized SPS decreased as the plasticizer concentration was added from 15–45%, which reflect the stiffness of the material is reduced. Glycerol plasticized film portrays the highest degree mobility of the polymer chain among other plasticizers.

Polymer films made from poly-(vinyl alcohol), low-density polyethylene, poly-(vinyl alcohol), or polybutylene are used for mulch manufacturing [61]. These films are altered in a specific manner, such that they are permitted to go through degradation only when the crop-growing period is complete, either through the aid of soil micro-organisms or through the addition of certain particulate matter, which promotes film cessation [31]. A similar trait was observed in polycaprolactone for making agricultural plant containers. These containers tend to biodegrade over a significant period of time, allowing tree seedlings to adequately sprout. Implementation of natural fibre as a promising alternative to synthetic fibre in the composite industry is seen to be increasing in past decades since the resulting bio composite exhibits favourable properties. The incorporation of natural fibres can further enhance the mechanical properties of materials and lead to the high possibility of substituting synthetic polymers.

Oliveira et al. [68] studied the application of a biodegradable tray utilizing starch and PLA blends coated with beeswax by flat extrusion, calendaring, and hot-pressing under variance of beeswax coating emulsion 1, 2, and 3 g wax/100 g solutions. The mechanical result shows beeswax concentration (BC) 1 g/100 g solutions showing the highest tensile strength that might be attributed to the better adherence of the coating that has improved material strength properties. The Young modulus value is reduced from BC1 to BC2 and BC3 that could be due to lower tensile value and reflect elongation at no distinct value. Water vapor permeability (WVP) is an important characteristic in a food tray application and BC1 shows the lowest WVP value as compared to others. The application of the food tray is possible and can be produced at a large scale due to good tensile result and less affinity toward moisture.

PBS has been exhibited as mulch film, packaging, and flushable hygiene products and is considered as a non-migrant plasticizer for polyvinyl chloride (PVC) [71]. Moreover, PBS is used in foaming and food packaging application. The relatively poor mechanical flexibility of PBS restricts to the applications of 100% PBS-based products. Nevertheless, this can be solved by combining PBS with PLA or starch to enhance the mechanical properties significantly, which promote properties more or less the same to that of polyolefin [70].

Utilization of natural fibre incorporated with advantages as compared to synthetic fibre in terms of tensile properties, less health hazard, acceptable insulating properties, low density, and less energy consumption [27]. A possible waste exploitation were practiced by many researchers, utilizing the cassava bagasse as a material product’s filler reinforced agent to enhance the material properties currently increasing. These by-products are found low in market value and can possess good properties such as a reinforcing agent and a wide range of application that can be implemented, such as production of organic acid, bio-degradable packaging, nano particles, nanofibers, ethanol, bio-fuel, lactic acid, and many more [76].

## 8. Conclusions

This paper conducts a general overview on biopolymers and the potential of starch-derived thermoplastics as substitutes for current petroleum-based plastic. Blending starch with other biopolymers was outlined as a viable alternative to overcome the shortcomings of native starch. However, the degree of compatibility between starch and other biopolymers extensively differs depending on the specific biopolymer. At present, mixing TPS and PLA offers significant advantages in terms of cost, properties, and biodegradability.

Regarding the global environmental issue, biodegradable material properties are vital and should be considered. Although starch/biodegradable blends are a good option for solving environmental issues, their mechanical properties often have an inverse relationship to their degradability. Incorporating natural fibres as fillers in the starch matrix can be another solution. Thus, optimising their mechanical properties requires further analyses.

## Figures and Tables

**Table 1:** Thermoplastic cassava starch composite.

Type of Starch	Type of Filler/Polymer	Potential Application	Ref.
Cassava	Kraft	Biodegradable tray with chitosan coating	[6]
Cassava	Orange, sugarcane, malt bagasse	Biodegradable tray, packaging material	[3]
Cassava	Cogon grass	Biodegradable material	[7,25]
Tapioca	Bamboo	Biodegradable ‘green’ plastic	[17]
Cassava	Sugar palm	Biodegradable polymer	[6]
Cassava	Grape stalks	Food packaging plastic	[20]
Cassava	Cassava bagasse	Food packaging plastic	[18]

**Table 2:** Thermoplastic corn starch composite.

Type of Starch	Type of Filler/Polymer	Potential Application	Reference
Corn	Sugarcane	Green material for packaging	[46]
Corn	Microalgae	Bioplastic, i.e., packaging, catering products, electronic devices	[47]
Corn	Cassava, ahipa peels and baggase	Bio-based composite	[19]
Corn	Talc nanoparticles	Bio-nanocomposite food packaging	[48,49]
Corn	Nanocrystalline cellulose	Bio-film and bio-nanocomposite	[50]
Corn	Sunflower seed fried oil	Potential natural plasticizer	[38]
Corn	PLA blends	Bio-degradable polymer	[51]
Corn	Cornhusk/sugar palm	Hybrid bio-composite	[52]

**Table 3:** Mechanical test result [46].

Test Type	GC (%)	FC (%)	Result
Tensile Test, σ	24–28	10–15	Highest σ value achieved at 35MPa.
Elongation, ε	24–28	5–15	Highest ε value achieved at 21.7%
Young Modulus, E	24–28	10–15	Highest E value achieved at 1433.8 MPa

**Table 4:** Thermoplastic sugar palm starch composite.

Type of Starch	Type of Filler/Polymer	Potential Application	Reference
Sugar palm	-	Biodegradable material	[1]
Sugar palm	-	Biodegradable packaging film	[32]
Sugar palm	Agar blends	Bio-based polymer	[55,56]
Sugar palm	Agar blends, seaweed	Bio-based polymer	[57]
Sugar palm	Sugar palm fibre	Bio-nanocomposite material, food packaging	[52,53,58]
Sugar palm	Agar blends, sugar palm fibre, seaweed	Hybrid bio-composite	[36]

**Table 5:** Advantages of natural fibres compared to synthetic fibre [29].

	Natural Fibres	Synthetic Fibres
Density	Light	Twice natural fibres
Cost	Low cost	Higher than natural fibres
Renewability	Yes	No
Recyclability	Yes	No
Energy Consumption	Low	High
Distribution	Wide	High
CO_2_ neutral	Yes	No
Health risk when inhaled	No	Yes
Disposal	Biodegradable	Yes, not biodegradable

**Table 6:** Applications of biopolymers.

Blends	Application	Reference
Starch/plasticizer	• Biodegradable packaging	[1]
	• Starch based film material	[32]
	• Disposable eating utensils	[38]
Starch/PVA	• Water-soluble laundry bags	[61]
	• Biomedical and clinical field	[66]
	• Replacement of polystyrene	[63]
Starch/PLA	• Biodegradable tray	[68]
	• Electronic devices, pharmaceutical	[62]
Starch/PBS	• Packaging materials, fisheryAutomotive	[61,65]
Starch/natural fibre	• Food packaging	[20,25]
	• Biodegradable material	[22,58,68]

## Data Availability

The data presented in this study are available on request from the corresponding author.
